# Exploring the Microbial Mosaic: Insights into Composition, Diversity, and Environmental Drivers in the Pearl River Estuary Sediments

**DOI:** 10.3390/microorganisms12071273

**Published:** 2024-06-23

**Authors:** Tal Zvi-Kedem, Maya Lalzar, Jing Sun, Jiying Li, Dan Tchernov, Dalit Meron

**Affiliations:** 1 Morris Kahn Marine Research Station, Faculty of Marine Biology, Leon H. Charney School of Marine Sciences, University of Haifa, Haifa 3498838, Israel; tal.zvi.kedem@gmail.com (T.Z.-K.); dtchernov@univ.haifa.ac.il (D.T.); 2 Bioinformatics Services Unit, University of Haifa, Haifa 3498838, Israel; maya.lalzar@gmail.com; 3 Department of Ocean Science, The Hong Kong University of Science and Technology, Hong Kong, China; jsunbk@connect.ust.hk (J.S.); jiyingli@ust.hk (J.L.); 4 Southern Marine Science and Engineering Guangdong Laboratory (Guangzhou), Zhuhai 519080, China

**Keywords:** sediment, microbiota, Pearl River Estuary, microbial markers, environmental drivers

## Abstract

River estuaries are dynamic and complex ecosystems influenced by various natural processes, including climatic fluctuations and anthropogenic activities. The Pearl River Estuary (PRE), one of the largest in China, receives significant land-based pollutants due to its proximity to densely populated areas and urban development. This study aimed to characterize the composition, diversity, and distribution patterns of sediment microbial communities (bacteria, archaea, and eukaryotes) and investigated the connection with environmental parameters within the PRE and adjacent shelf. Physicochemical conditions, such as oxygen levels, nitrogen compounds, and carbon content, were analyzed. The study found that the microbial community structure was mainly influenced by site location and core depth, which explained approximately 67% of the variation in each kingdom. Sites and core depths varied in sediment properties such as organic matter content and redox conditions, leading to distinct microbial groups associated with specific chemical properties of the sediment, notably C/N ratio and NH4+ concentration. Despite these differences, certain dominant taxonomic groups were consistently present across all sites: Gammaproteobacteria in bacteria; Bathyarchaeia, Nitrososphaeria, and Thermoplasmata in archaea; and SAR in Eukaryota. The community diversity index was the highest in the bacteria kingdom, while the lowest values were observed at site P03 across the three kingdoms and were significantly different from all other sites. Overall, this study highlights the effect of depth, core depth, and chemical properties on sediment microbiota composition. The sensitivity and dynamism of the microbiota, along with the possibility of identifying specific markers for changes in environmental conditions, is valuable for managing and preserving the health of estuaries and coastal ecosystems.

## 1. Introduction

River estuaries stand as intricate intersections between terrestrial and marine ecosystems characterized by dynamic conditions perpetually influenced by various natural processes. These transitional zones are subject to climatic fluctuations such as changes in temperature and precipitation, which in turn influence the influx of freshwater, terrestrial runoff, and incoming tides. These fluctuations may alter oxygen levels, salinity, pH, and other environmental parameters [1,2,3,4,5]. The confluence of these factors establishes a complex environment that harbors enriched nutrients, mainly carbon and organic matter [6,7]. The sources of these nutrients encompass river runoff, land surface runoff, and atmospheric deposition [8]. These areas, which are often characterized by high population density, are additionally exposed to high anthropogenic activity (including domestic sewage, industrial wastewater, agriculture fertilizer, and marine culture), which impacts the marine ecosystem through terrestrial runoff [9,10]. The resulting variability in the composition and quantity of materials contributes to a dynamic environment characterized by fluctuating environmental conditions. 

Among these estuaries, the Pearl River Estuary (PRE), China’s second-largest estuarine system by discharge volume, stands as a poignant example of environmental stress. The large population living near it, the massive regional economic growth, and rapid urban development have led to the excessive release of waste into the estuary [7,8,11]. These waste flows introduce high loads of nutrients and organic matter from both industrial and agricultural sources [8,12]. The discharge of the river changes between seasons (dry and wet seasons) which affects the biological, physical, and chemical processes, such as flow of water volume, turbulent dispersion, nutrient amount, oxygen, salinity, algal blooms, etc. [13,14,15]. For example, during summer, high concentrations of nutrients (leading to eutrophication) may cause excessive algae blooms and low O_2_ concentrations in bottom waters, even producing hypoxic patches [16,17]. 

Due to its ecological complexity and importance, the PRE has been the focus of numerous studies on different facets of this ecosystem. Studies have focused on aspects ranging from organic matter [7,18] and fatty acid composition [19] to heavy metals [20], PFAS concentrations [21], and pollutant concentrations along the estuary. The values of these parameters and other pollutants are used to monitor and follow the environmental impact and the changes. Another critical aspect of the environment is the microbiota. Due to their short generation time, high functional diversity, and sensitivity, the microbial communities are dynamic and can rapidly respond to physical, chemical, or biological changes and therefore may serve as important indicators of environmental changes [22,23]. Several articles on PRE have shown the effect of various parameters such as seasonality, salinity, and pollution on the composition and diversity of the microbiota [24,25,26,27]. 

In contrast to highly variable water samples, even diurnally, the sediments are more stable and therefore more probable to produce robust indicators for marine health or the presence or persistence of particular pollutants. The sediment which serves as a reservoir and a sink for various compounds, plays an essential environmental role in nutrient cycling and biogeochemical processes, due to its capacity to store or release different compounds from or to the water column [28,29]. Within the intricate framework of estuarine ecosystems, sediment microbial communities assume a crucial role in governing benthic biological processes. These communities are responsible for many global biogeochemical activities, including pivotal cycles such as nitrogen, carbon, phosphorus, and sulfur [30,31]. Additionally, they form the foundational layer of aquatic food chains, contributing to organic matter demineralization and the degradation of pollutants [32,33]. However, the functioning of these microbial communities is notably susceptible to the impacts of changing environments [34]. The variability in the conditions of the estuarine area has resulted in microbial communities, particularly in surface sediments, displaying richer composition and biodiversity compared to marine ecosystems [35,36]. 

This study examined the microbiota at the outskirts of the PRE, aiming to describe key drivers shaping the microbiota structure. To provide a comprehensive description of the microbiota, bacteria, archaea, and Eukaryota were considered with respect to commonality and differences in responses to various environmental parameters. The study focused on sediment microbiota, different sites, and core depths, a choice based on the consideration of future endeavors to identify microbial markers related to the health status of this marine environment.

## 2. Methods 

### 2.1. Study Site and Sampling Procedure

**Figure 1:**
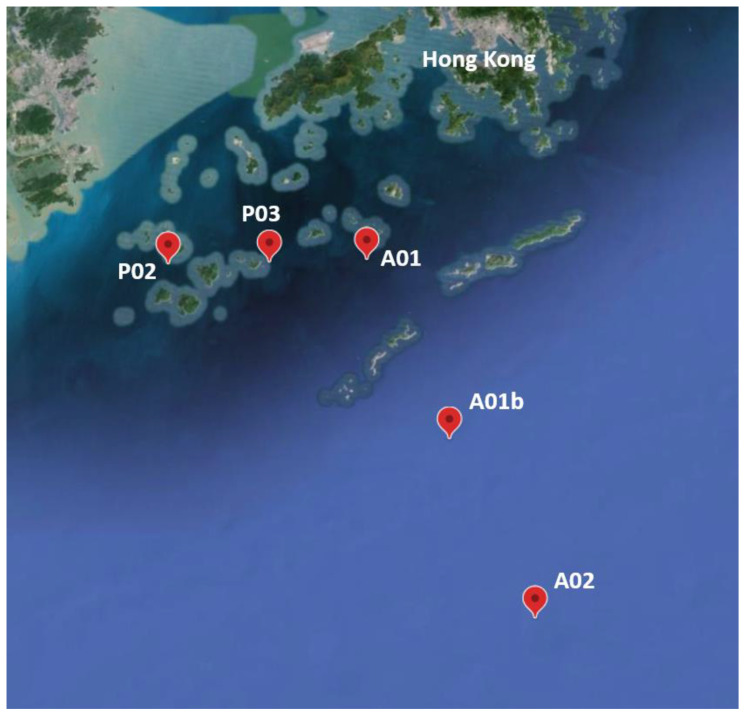
Map of the sampling sites, next to the Pearl River Estuary. Site coordinates are presented in Table S1.

Sediment samples were collected across the Pearl River Estuary (PRE) and adjacent shelf during July 2021 on the Research Vessel Haike 68. The sampling sites were in a depth range of 16 to 63 m and comprised the following sites: P02, P03, A01, A01b, and A02 (Figure 1, Table S1). Sites A01b and A02 are deeper, exposed to the open sea, and considered as marine sites. In contrast, sites P02 and P03 are shallower and located near the river estuary. Site A01 is an intermediate point in terms of depth and location. On the one hand, it is close to the estuary of the river but on the other hand, it is deeper and more exposed to the open sea than P02 and P03. Sediment cores were taken using a Uwitec corer with an 86 mm inner diameter and 60 cm length. Sediment cores were sectioned onboard with a vertical resolution of 1–5 cm (1 cm at the surface and 5 cm at the bottom), with 9 slices per core in total. Thus, for the 5 sites, we obtained a total of 45 samples. The nine slices for each core were classified into three depth sets: UP (1–3 cm), MED (5–9 cm), and DEEP (10–25 cm) (Table S2). The sediment samples for analysis of DNA and porosity were sectioned in air; sediment samples for analysis of solid and solute chemical substances were sectioned in a glove bag filled with nitrogen gas to avoid oxidation. To analyze the soluble substance, porewater was extracted using Rhizon samplers with 0.1 μm pore size (Rhizosphere Research Products, Gelderland, The Netherlands). Samples for analysis of dissolved Fe were preserved with 5% (*v*/*v*) nitric acid. Other porewater and sediment samples were stored at −20 °C for further analysis. 

### 2.2. Environmental Parameters Analysis

We analyzed environmental parameters, including the concentrations of dissolved oxygen (O_2_), ammonium (NH_4_^+^), dissolved iron (Fe_diss_), sediment porosity, total nitrogen (TN), and particulate organic carbon (POC, Table S2). To measure POC and TN, ~0.5 g of dried sediments were mixed with hydrochloric acid (0.5 N, 1 mL) overnight to dissolve the particulate inorganic carbon (PIC). The samples were then dried at 60 °C and measured for POC and TN using an elemental analyzer (EA, Euro EA3000, Enrovector). Dissolved oxygen was measured using a micro-profiling system assembled with an oxygen sensor (Unisense, Aarhus, Denmark). Fe_diss_ concentrations were determined spectrophotometrically using a Ferrozine method [37]. Ammonium concentrations were determined by the indophenol blue method [38]. Sediment porosity was calculated by water content and dry sediment density, which were measured by the weighing method and pycnometers separately [39]. POC and TN in sediment were treated with hydrochloride acid to remove inorganic C prior to measurement using an elemental analyzer (EA, Euro EA3000, Eurovector, Milan, Italy) coupled to an Isotope Ratio Mass Spectrometer (IRMS, Nu Perspective, Nu Instruments, Wrexham, UK). The data for O_2_, POC, and TN and extended materials and methods for these measurements were reported previously in a thesis work [40].

### 2.3. DNA Extraction, PCR Amplification, and Amplicon Sequencing 

The methods in Section 2.3 and Section 2.4 are based on the methods described in our previous articles [41,42]. From each slice, DNA was extracted utilizing the DNeasy powerSoil Kit (Qiagen, Valencia, CA, USA) following the manufacturer’s instructions. Every step of the sample preparation process contained the appropriate controls (no input reactions). For bacteria and Eukaryota, primers were used for PCR amplification of SSU rRNA gene fragments, targeting the V4 regions of microbial small subunit ribosomal RNA genes as described in The Earth Microbiome Project (bacteria, CS1-515F: ACACTGACGACATGGTTCTACAGTGCCAGCMGCCGCGGTAA and CS2-806R: TACGGTAGCAGAGACTTGGTCTGGACTACHVGGGTWTCTAAT (~290 bp); Eukaryota: CS1-Euk1391F: ACACTGACGACATGGTTCTACAGTACACACCGCCCGTC and CS2-EukBr-R: TACGGTAGCAGAGACTTGGTCTTGATCCTTCTGCAGGTTCACCTAC (~200 bp)) [43,44]. For archaea, the primer pair used was published in Takahashi et al. (2014) [45] (CS1-ARC344F: ACACTGACGACATGGTTCTACAACGGGGYGCAGCAGGCGCGA and CS2-ARC806R: TACGGTAGCAGAGACTTGGTCTGGACTACVSGGGTATCTAAT (~400 bp)). The primers contained 5′ common sequence tags (known as common sequences 1 and 2, CS1 and CS2 [46]. Amplicons were generated using a two-stage PCR amplification protocol. Cycling conditions for the first stage PCR were 95 °C for 5 min, followed by 28 cycles of 95 °C for 30 s, 55 °C for 45 s, and 72 °C for 60 s. Library preparation (including sample barcoding and addition of PhiX DNA spike-in control), pooling, and sequencing (Illumina (Fluidigm, South San Francisco, CA, USA; Item# 100-4876) MiSeq (archaea) or MiniSeq (bacteria and Eukaryota) were performed at the Research Resources Center (GRC) within the Research Resources Center (RRC) at the University of Illinois at Chicago (UIC). The data are accessible via GenBank under the SRA accession number: PRJNA1031164. 

### 2.4. Sequence Data Processing

Sequence data were processed using the Dada2 pipeline [47]. Quality trimming, sequence error correction, amplicon sequence variants (ASVs) inference and quantification, and error model estimate were carried out independently for every sequencing run. The ASVs and count tables of all runs were merged and the suspected chimera were removed. For each ASV, taxonomy was inferred by alignment to the Silva non-redundant small subunit ribosomal RNA database (v138) using the Dada2 command ‘assignTaxonomy’, setting the minimum bootstrap value to 80%. For Eukaryota ASV sequences, taxonomic inference was also achieved by the last common ancestor method (LCA) in MEGAN (MEGAN6, community addition) using the input results of BLASTn against the NCBI nt database with 50 best hits (LCA parameters: minimum score = 100, an e-value < 10^−9^ and a coverage > 80%). Table S6a–c presents the raw count tables and ASVs inferred taxonomy. 

### 2.5. Community Structure with Environmental Variables (metaNMDS) 

We used non-metric multidimensional scaling (NMDS) to explore the similarity in community structure between the different samples. We repeated the following analysis for each combination of taxonomic group and aggregation level. We first filtered the OTU and kept only those with a minimum of 5 total individuals. We applied a Wisconsin square root transformation on the raw abundance data before calculating the Bray–Curtis dissimilarity between each sample pair. Next, we selected 33 samples out of 45 that had complete environmental data for the selected variables (CoreDepth, NH_4_^+^, Porosity, TN, POC, C/N, and O_2_; Table S2). The reduced dataset included at least 5 samples from each site. Core depth categories were represented by at least 7 samples. We then fitted a metaNMDS model using the metaMDS function of the vegan R package [v2.5.7] [48]. The lengths of the vectors are scaled by R^2^ of the multiple regression and are relative. We used vegan’s envfit function (999 permutations) to determine the relative contribution of environmental variables to the separation of the communities along ordination axes. The direction of the environmental vectors is based on multiple regression against the ordination axes, while vector lengths are scaled by the R^2^ of the regression (Table S3). To examine the contribution of the environmental factors (site and core-depth) to the variation in microbiota composition in model samples, permutational analysis of variance (PERMANOVA) was performed (command ‘adonis’ in R package ‘vegan’ [v2.5.7]) based on Euclidean distances of log-ratio transformed counts. The Shannon index was computed for each of the three datasets by subsampling the raw counts to the smallest library size (using the “diversity” function in the R package “vegan”). Aligned rank-transformed (ART) ANOVA was used to examine variations in the Shannon index as a function of the environmental factors.

### 2.6. Biomarker Identification (LDA and LDM)

Linear discriminant analysis effect size (LEfSe) analysis was selected to calculate differential abundance and identify putative biomarkers. This technique works well for identifying the features, in this case, ASVs, that are most likely to account for the observed variations across factor levels [49]. LEfSe was performed using the MicrobiotaProcess R package [v3.16] [50]. LDA-based effect size analysis is widely implemented in microbiota analysis and can provide valuable insights into the importance of different features in discriminating between different groups, as well as identify the most informative features for classification tasks. We used the CSS (Cascading Style Sheets) normalized data as input and performed the analysis separately once to identify biomarkers and differential abundances for sites, and once for core-depth categories. In both cases, we set the LDA effect size threshold to 1.25 and estimated the Benjamini and Hochberg (1995) [51] false discovery rate for multiple comparisons to correct the *p*-value and reduce false positives. We explored the relative abundances of the identified markers and plotted them on cladograms based on taxonomic relatedness. Linear decomposition model (LDM) analysis [52] was applied for the detection of ASVs with relative abundance significantly associated with the chemical properties of the sediment. LDM was performed using the R package ‘LDM’ (version 5.0). Significant differential abundance was considered for q value < 0.05 in the ‘omni3’ test. 

## 3. Results

### 3.1. Microbial Composition and Environmental Drivers 

**Figure 2:**
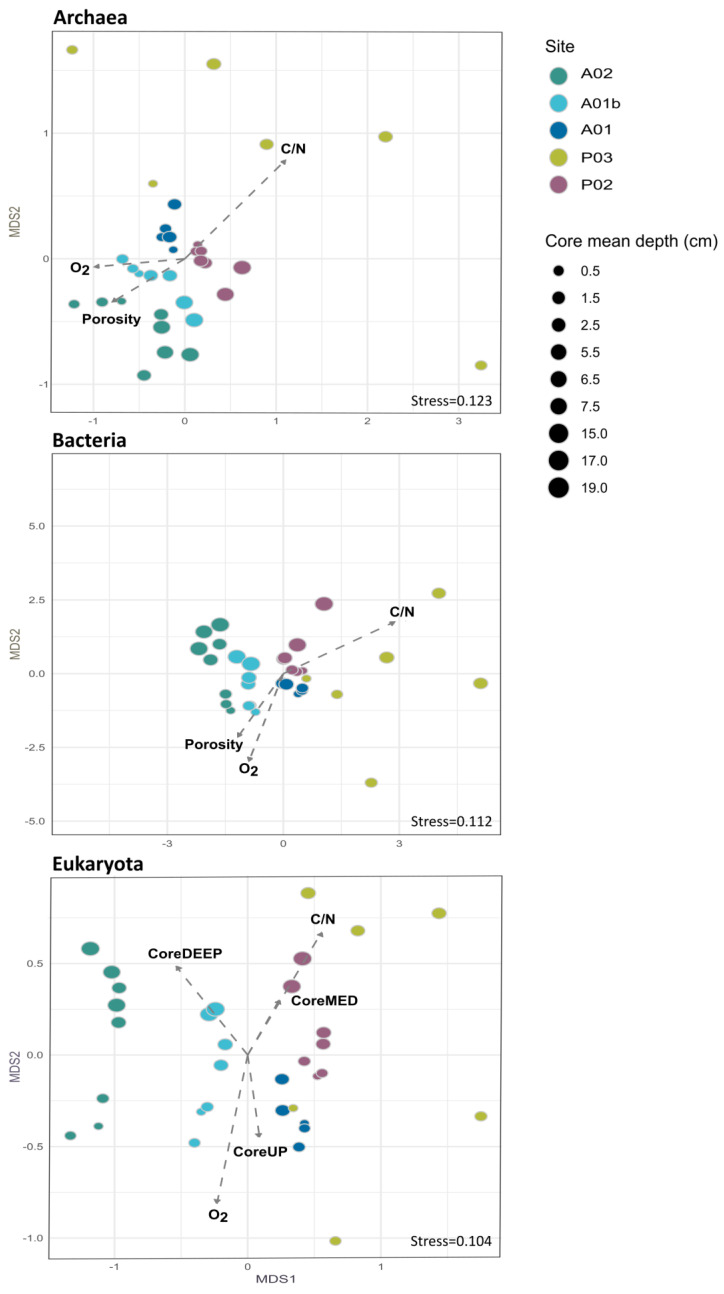
Non-metric multidimensional scaling analysis (NMDS) of the microbial communities and the relationship between environmental variables and the NMDS ordination axes. The environmental variables vector as identified by the envfit analysis (*p*-values and R^2^ of multiple regressions against the ordination axes are shown). Of all the measured environmental variables (Table S2), only those that were significant in each kingdom are shown (Table S3).

**Figure 3:**
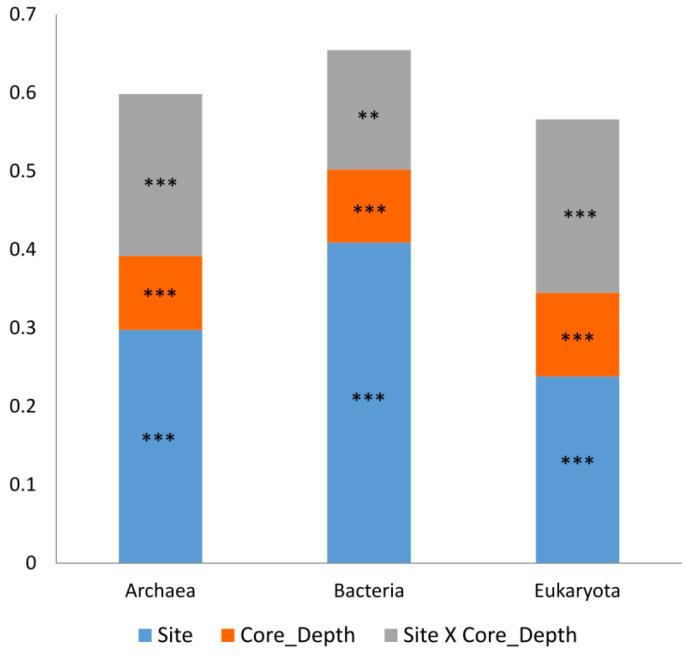
Effect of the site and the core depth on sediment microbiota. PREMANOVA factorial test results. ** *p* < 0.01; *** *p* < 0.001.

The study characterizes the composition of sediment bacteria, archaea, and eukaryotes at five sampling sites along a depth gradient from the Pearl River Estuary to the South China Sea (Figure 1, Table S1). The microbiota was compared between sampling sites and core depth and in relation to measured environmental parameters (Table S2). In total, 2,038,667 (993,589 archaea, 782,063 bacteria, and 263,015 Eukaryota) sequences from 45 sediment samples were analyzed. Following the sequence analysis, 15,220 ASVs were identified (5478 archaea, 7215 bacteria, and 2527 Eukaryota). We first examined the similarity in microbiota composition among samples. For this purpose, non-metric multidimensional scaling analysis (NMDS) based on Bray–Curtis dissimilarities was calculated for each kingdom. Similar trends were detected in all the kingdoms (Figure 2). The first axis of the ordination was related to the sampling site and the samples were organized (left to right) separating the marine sites and estuary sites. Additionally, for each site, samples from deep core layers were separated from the upper ones. Notably, the variance among samples from site P03 was higher compared to other sites across all kingdoms (Figure 2). Multiple regression coefficients between environmental factors and NMDS ordination axes were calculated, which indicate some common trends among kingdoms (Figure 2; Table S3). Higher O_2_ concentration and, for bacteria and archaea, porosity were related to negative ordination values, where marine sites were positioned, and higher C/N ratio values were related to positive ordination values, associated with estuary samples. In order to test the contribution and significance of site and core depth and their interaction on variation in microbiota composition, a permutational analysis of variance (PERMANOVA) test was applied. The site, core depth, and their interaction were all significant and explained about 67% on average of the variation in each kingdom, where the site was the main factor and explained 33% in eukaryotes, 41% in archaea, and 49% in bacteria (Figure 3 and Table S4). Pair-wise tests among sites showed significant differences between all the sites (Table S5). 

**Figure 4:**
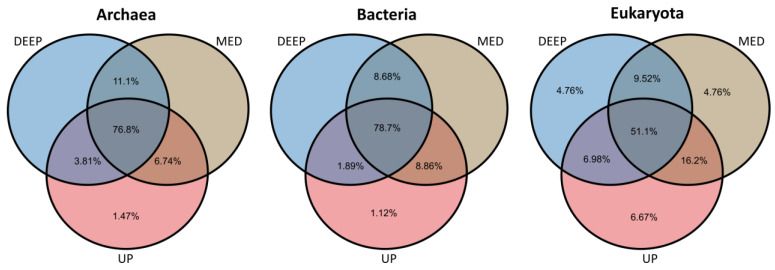
Venn diagrams indicating the distribution of unique and shared microbial ASVs between the core depths (DEEP, MED, and UP). ASVs with prevalence >10% of samples were included. Only values above 1% are shown.

Most of the prevalent ASVs detected (above 10% of samples) appeared at all core depths (UP, MED, and DEEP), including 78.7%, 76.8%, and 51.1% in bacteria, archaea, and eukaryotes, respectively. Unique ASvs of the core depths were negligible and ranged between 0 and 1.5% in bacteria and archaea and up to 6.7% in eukaryotes. In all cases, the UP layer had the highest percentages of unique ASVs in all kingdoms. As may be expected, higher common ASVs were observed between MED and up/deep depths compared to common ASVs between UP and DEEP depths (Figure 4). A comparison of the ASVs between the five sites showed only 7.9% of bacteria, 15% of archaea, and 22% of eukaryotes. ASVs were common to all sites (Figure S1). The unique ASVs in archaea and bacteria were 3.2% and 4.56% in the A02 site, while less than 1% and 2% in the rest sites (respectively). The percentages of unique sequences were higher in eukaryotes at the different sites (2.5–5.7%), except for the P03 site, which had less than 1%. 

### 3.2. Sediment Microbial Taxonomy and Diversity

**Figure 5:**
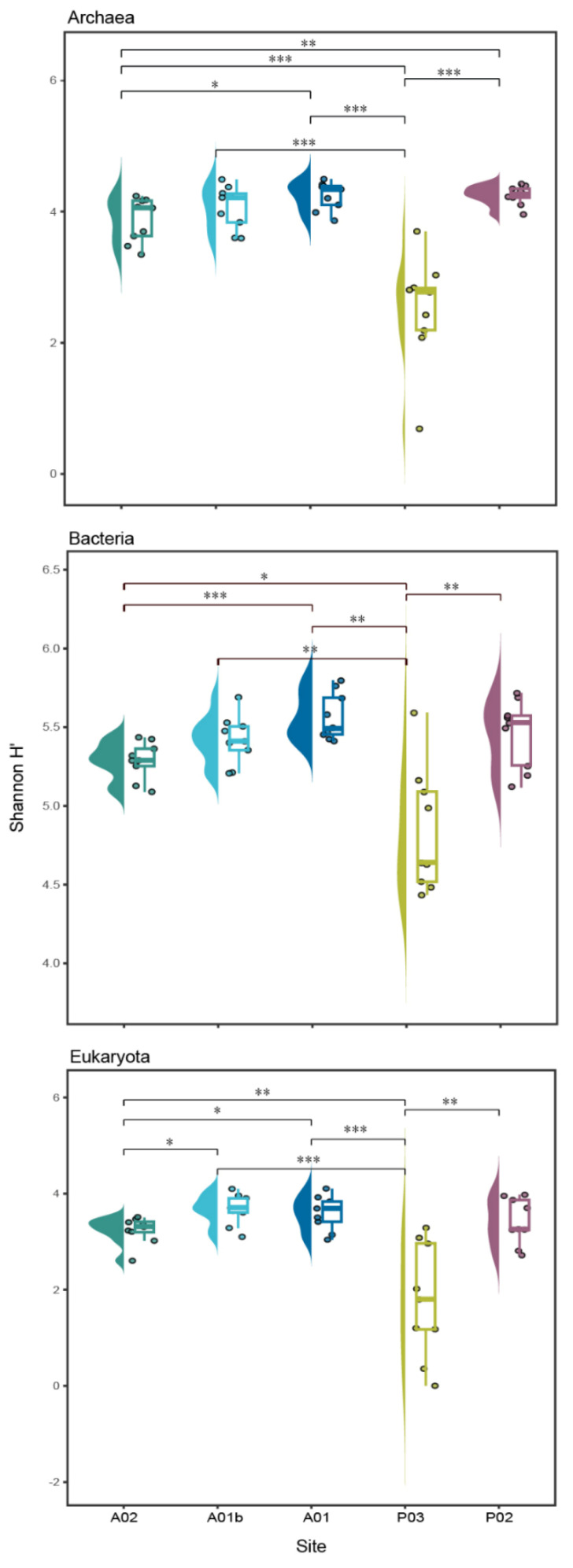
Boxplot presenting the distribution of Shannon H’ index of diversity within each site at each kingdom. * *p* < 0.05; ** *p* < 0.01; *** *p* < 0.001.

**Figure 6:**
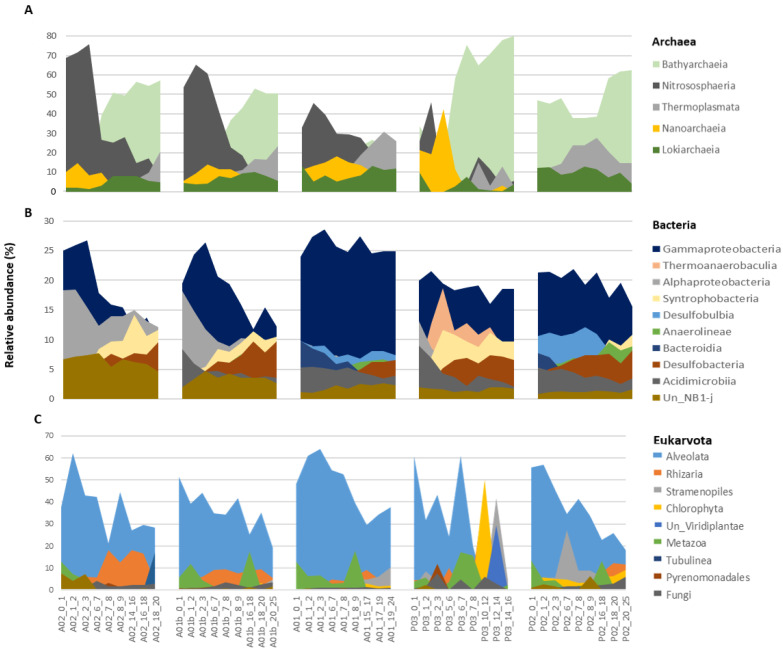
Composition and structure of sediment microbiota in the sampled sites. (**A**) Archaea class-level composition. (**B**) Bacteria class-level composition and (**C**) Eukaryota TAX 2 level. The groups above 6% relative abundance are shown.

When comparing the diversity index (Shannon H’) between the transect sites, the highest values were observed in the bacteria kingdom. The lowest values of Shannon were indicated at site P03 and were significantly different from all the other sites in the three kingdoms (Figure 5). In contrast to the sites, no significant differences were observed between the core depths (UP, MED, and DEEP), apart from a significant difference in Shannon between UP and DEEP only in archaea (Figure S2). The main taxonomic groups of archaea included 5 main classes, namely Bathyarchaeia, Nitrososphaeria, Thermoplasmata, Nanoarchaeia, and Lokiarchaeia, which appeared in all sites and together constituted over 91% of population composition on average. An opposite trend is seen between the sites. At the estuary sites (P03 and P02), Bathyarchaeia were highly dominant (average ~50%), while Nitrososphaeria relative abundance was lower (average ~10%); at the marine sites (A02 and A01b), Nitrososphaeria dominated and the relative abundance of Bathyarchaeia was lower. In addition, there were some trends observed along the core depth; for example, in the sites A02, A01b, and A01, the relative abundance of Nitrososphaeria decreased with the core depth, while the relative abundance of Thermoplasmata increased (Figure 6A, Table S5). In bacteria, Gammaproteobacteria was the main dominant class in all sites. At the marine sits, the relative abundance of Proteobacteria (classes Gammaproteobacteria and Alphaproteobacteria) decreased with core depth while the relative abundance of Syntrophobacteria, Desulfobacteria, and Anaerolineae classes increased. In P03, in contrast to other sites, the Thermoanaerobaculia was the second dominant class, while in P02 and A01, the Desulfobulbia was the dominant class. These presented classes constituted over 70% on average of the bacterial composition (Figure 6B, Table S5). Identification of Eukaryotic ASVs at a high taxonomic resolution was more challenging. A high percentage of the ASVs (~36%) were identified only at the kingdom level (i.e., unclassified Eukaryota). Most of the identified Eukaryotic ASVs belonged to the SAR group (i.e., Alveolata, Stramenopiles, and Rhizaria) (average of 48% of all the samples), with Alveolata as the dominant group (40% from the SAR). Rhizaria were detected mainly in the marine samples, while Stramenopiles were observed in the estuary sites (Figure 6C, Table S5).

### 3.3. Microbial Markers

**Figure 7:**
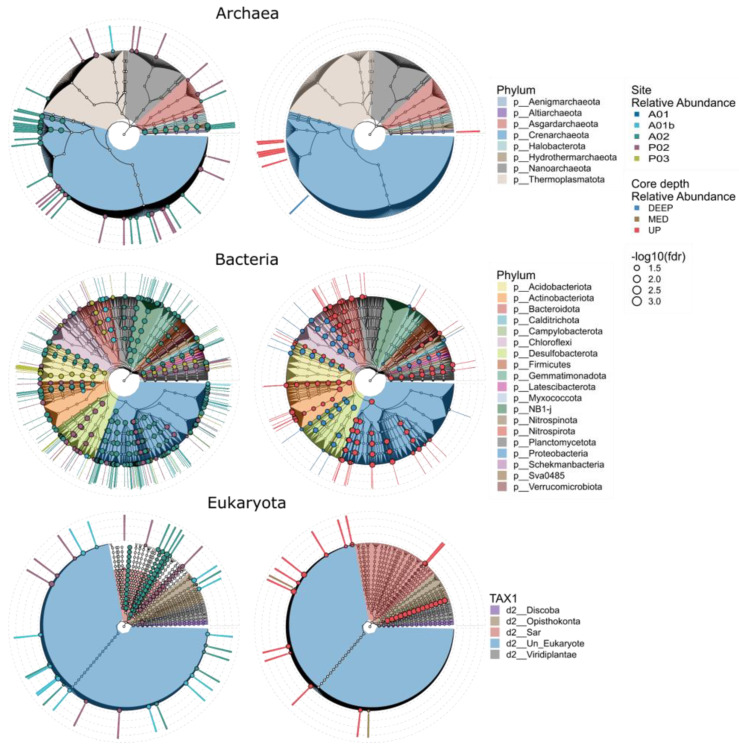
Microbial markers of the site and core depth are presented by a radial cladogram based on the hierarchical taxonomy, colored by phyla (TAX1 for eukaryotes) with the relative abundance in each site and core depth. The markers were identified by the linear discriminant analysis effect size (LEfSe) methods (BH adjusted *p* < 0.05, LDA > 2).

**Figure 8:**
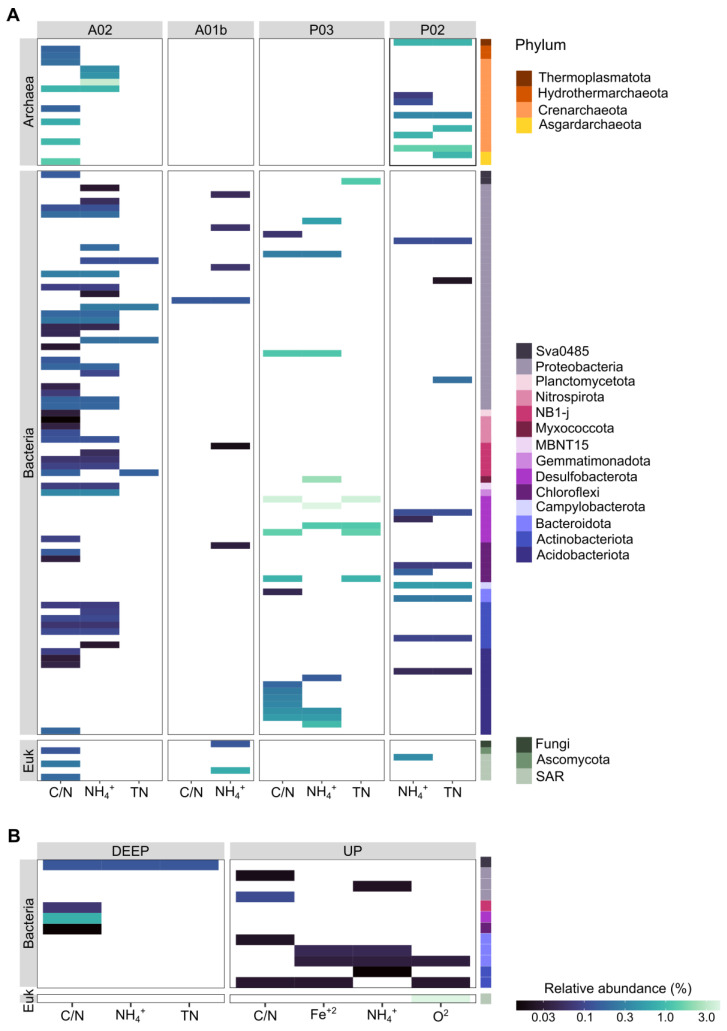
Correspondence between site (**A**) or core depth (**B**) specificity and sediment chemical parameters. Marker populations (ASVs) for each site were determined by linear discriminant analysis (LDA) effect size (LefSe). In parallel, significant effects of sediment physiochemical properties (i.e., C/N, TN, NH_4_^+^, and O_2_) were examined by linear decomposition model (LDM) analysis. ASVs significant for both site/core depth and at least one chemical parameter are presented. The colors in the legend refer to the two parts of the figure.

We used Linear discriminant analysis effect size analysis (LEFSe) to search ASVs that exhibit differential abundance patterns in regard to either site identity or core depth. Microbial markers were identified in all kingdoms and included 42, 180, and 32 site markers and 8, 50, and 14 core depth markers for archaea, bacteria, and Eukaryota, respectively (Figure 7 and Table S7). Most of the bacterial markers found were associated with site A02, the farthest marine site (Figure 7 and Figure 8). The markers of archaea were found mainly in the UP layer of the core and belong to the family Nitrosopumilaceae. According to sites, markers belonging to the class Bathyarchaeia were found in sites P02 and A02 sites. Additional markers of A02 belonged to the family Nitrosopumilaceae and the order Hydrothermarchaeales. In Eukaryota, markers were identified mainly in the UP core layer and for sites P02, A02, and A01b, while most of the ASVs belonged to SAR. In contrast to the previous kingdoms, bacterial markers were found in the DEEP layer as well. For example, in the UP core layer, representatives belonged to phylum Actinobacteriota, Bacteroidota, and Proteobacteria (Gammaproteobacteria and Alphaproteobacteria), and in the DEEP layer, they belonged to Chloroflexi, Desulfobacterota, and Acidobacteriota. Markers were found mainly in sites A02 (e.g., Proteobacteria (Alphaproteobacteria and Gammaproteobacteria) and NB1-j), P03 (Acidobacteriota), and P02 (Proteobacteria and Desulfobacterota). In order to identify ASVs significantly associated with chemical properties (e.g., C/N, TN, NH_4_^+^, and O_2_), a linear decomposition model (LDM) analysis was performed. Figure 8 shows the correspondence between site/core depth specificity and sediment chemical parameters. We found that 153 ASVs in total correlated with sites 23, 124, and 6 (archaea, bacteria, and Eukaryota, respectively) and with a core depth of 12 markers for bacteria and 1 for Eukaryota. Common ASVs were identified in four sites and included only the three parameters: C/N, NH_4_^+^, and TN. Most markers were found in site A02 in all kingdoms and were correlated mainly with C/N and NH_4_^+^. ASVs shared between archaea and chemical parameters were found only in sites A02 and P02, with most belonging to Crenarchaeota. Site P03 included only bacterial markers with the highest relative abundance, mainly class Desulfobacteria. Markers related to the core depth were identified in the upper (mostly) and deep layers and were mainly correlated with C/N and NH_4_^+^. In contrast to the site’s markers, we found the upper layer markers that are related to the parameters O_2_ and Fe^+2^ as well. The most significant markers were Desulfobacteria with C/N in the deep layer and SAR correlation with O_2_ in the upper layer.

## 4. Discussion

Microbes are essential, inhabiting every estuarine environment, water, and sediment and carrying out biogeochemical processes and ecological functions that affect and shape the environment. Previous studies of the PRE area examined the chemical and physical conditions in the water column and sediment including seasonal variations [14,53,54]. The difference between the hot and wet summer compared to the cold and dry winter is expressed in the differences between the physicochemical and hence the biological parameters (as described Zhang by et al., 2023 [24]). The dry winter season, characterized by low river discharge and low water column stratification, limits the extent of the PRE horizontal plume and reduces hypoxic events [14,55]. The samples in this study were collected during the summer season when the amount of nutrients reaching the PRE is high, leading to high organic load and hypoxic conditions [14,55,56]. Our study describes the composition and diversity of PRE sediment microbiota of the three kingdoms in relation to the site, core depth, and physicochemical environmental parameters. The chosen sites differed in their proximity to the mouth of the river and their bottom depth, and therefore subject to different levels of disturbance leading to the formation of different physicochemical conditions, affecting the characteristics of the sediment core layers. 

The locations of the sites (nearshore to offshore) and core depths were the two dominant drivers separating the microbial communities demonstrating a strong link between the community structure and their biogeochemical functions. The first axis of ordination of the NMDS analysis appears to be related to the locations of the sampling sites (Figure 2), which explains the variation in the three kingdoms by using the PERMANOVA test (Figure 3). As heterotrophic processes are fueled by organic matter, the amount of organic matter deposited on the seafloor would also affect the locations of the redox zones. The nearshore stations close to the estuary (P02 and A01) had higher organic carbon contents in the surface sediments compared to the offshore sediments (A01b and A02) (Table S2). In the marine stations (A01b and A02), the values NH_4_^+^ and Fe on surface sediment were lower compared to the estuary stations (P02 and A01) as reported by Huang et al. (2021) [57] in PRE as well. The C:N ratios, which are indicators of the freshness of the organic matter [58,59], also suggest that the quality of organic matter separates the microbial community structures (lower values indicating fresher organic matter that has a planktonic origin, and higher values indicating relatively refractory and terrestrial origin) (Table S2, Figure 2). The site P03, however, is an outlier, with remarkably low organic carbon content and porosity (Table S2). The sediments there were a mixture of sandy and muddy sediments, which suggest that the sediments have been disturbed (non-steady state sedimentation). This might explain the strong dissimilarity of the microbial communities at different core depths of site P03 (Figure 2), which were also significantly different from all the other sites (Figure 5) (see discussion later). 

The second axis of the ordination in the NMDS analysis is likely related to core depth (Figure 2), consistent with the vertical zonation of microbial redox reactions. Microbial heterotrophic reactions typically follow an order of the energy yield of the reaction [60]: aerobic respiration (using O_2_) is the most favorable and thus would occur in the surface sediments, below which O_2_ is depleted, Fe manganese and iron reductions, denitrification, sulfate reduction, and methanogenesis would occur one below another, until all reactive organic matter is exhausted. This explains the decrease in O_2_ and POC with core depth (aerobic respiration) and the production of Fe_diss_ and NH_4_^+^ in the anoxic sediment from the degradation of organic matter, separating the microbial communities of different core depths (Figure 2). 

These two factors, site and core depth, were indeed the key drivers influencing the composition explaining 56–65% of microbiota variance among samples for all three kingdoms (Figure 3 and Table S4). Previous studies that examined the effect of site depth and core depth of marine sites (different regions and depths) also found site and core depth as the main drivers [41,61]. However, the percentages of explained variance in those studies were much lower, indicating a very strong impact of the river mouth on the nearby sites, including the marine sites. The fact that core depths and locations (related to environmental gradient) affect the microbial composition suggests a tight link between microbes and their biogeochemical environments. Therefore, microbial taxa might provide insights into the variability of biogeochemical reactions in the sediments, which are driven by environmental gradients.

The depth of O_2_ penetrations (OPD) into marine sediments is among the most important parameters determining sediment biogeochemical processes. It controls the vertical zonation and rates of various organic matter mineralization pathways [62,63], organic carbon reactivity, and the fluxes of a dissolved substance across the sediment–water interface [39,64]. In sediments, oxygen continues to be consumed and is typically depleted from within a few millimeters in coastal sediment to several centimeters in pelagic sediments [64,65]. The anoxic sediments below the OPD allow diverse anaerobic heterotrophic carbon mineralization processes (e.g., denitrification, manganese reduction, iron reduction, sulfate reduction, and methanogenesis) and chemoautotrophic reactions (e.g., anaerobic methane oxidation) [66,67]. Previous studies have shown how a change in the oxygen level causes a shift in the composition of the microbial communities [68,69,70]. In this study, oxygen conditions varied greatly between the upper and lower core layers but also between the different sites (Table S2). Our results suggest that oxygen concentration has a greater effect on the upper sediment layer and the marine sites, which led to a significant marker presence of eukaryote representatives (Bacillariophyta and Alveolata), aerobic archaea (ammonia-oxidizing archaea (AOA)), Nitrosopumilaceae, and representative bacteria of Acidimicrobiia in this layer (Figure 7), which is also related to oxygen conditions in previous studies [69,71]. Additional bacterial markers of the deeper layer represented anaerobic bacteria including Anaeromicrobium, Desulfobacteria, and facultative bacteria belonging to Gemmatimonadota [72]. 

Our microbial community results also provide insights into the sediment nitrogen cycle. Nitrososphaeria (95% of the ASVs belonging to order Nitrosopumilales) were highly abundant in the PRE sediments [73,74,75], especially at the marine sites with a decreasing trend in correlation with the core depth [76]. Nitrososphaeria are renowned for their aerobic ammonia oxidation (AOA) activity in soils and marine environments [77]. Indeed, Nitrososphaeria relative abundance decreased with core depth (Figure 6), in parallel with loss of oxygen, which measured zero in all samples below 7 mm and (in most samples below 3 mm, Table S2 and Zhou, 2022 [40]). However, the relative abundance of Nitrososphaeria remained highly dominant in most samples at the anoxic core depths (Table S6a). As we have sampled DNA, we should consider that the source of the Nitrososphaeria at an anoxic depth sediment may be residual following the deposition of the sediment on top of older layers. Conversely, considering their high dominance, these Nitrososphaeria may represent species not obligatory to AOA. 

ASVs of the Desulfobacterota (classes Desulfobulbia and anaerobic Syntrophobacteria), sulfate-reducing bacteria (SRB) which are important in the sulfur cycle, were identified as markers for both estuary sites. Desulfobacteria increased with the core depth at all sites, which is consistent with the classic redox sequence that sulfate reduction would occur in deep sediments where other oxidants are exhausted [12]. In anoxic environments, such as estuarine sediments, SRBs are major contributors to carbon and sulfur cycles [12,78,79]. The fact that Desulfobacteria abundance is similar across the salinity gradient suggests salinity (sulfate concentrations in the overlying waters) is not the major control of sulfate reduction in the deeper sediments (Figure 6). Additionally, Desulfobacteria markers were correlated with C/N, NH_4_^+^, and TN parameters in the estuary sites (Figure 8 and Table S8). These results support the hypothesis that Desulfobacteria may be a good indicator of disturbed habitat (such as aquaculture) or environmental degradation [61,80,81]. Gammaproteobacteria followed by Alphaproteobacteria (in marine sites) or Desulfobacteria (in estuary sites) were dominant in the sediment samples, known as the main components of the marine sediment [41,82,83] and estuary area [84,85]. The dominance of Gammaproteobacteria was reported in coastal and estuarine water samples as well [86,87]. 

Among the sites, P03 was most divergent from all other sites in terms of composition and diversity (Figure 2, Figure 5 and Figure 6). At site P03, the microbial diversity in all kingdoms was significantly lower. A decrease in microbial diversity could indicate critical disturbance as previously described [88,89]. Variation in composition between core depths for this site was markedly higher than in other sites (Figure 2). Additionally, marker populations of this site were characterized by high relative abundance (Figure 8). Surprisingly, values of the main chemical parameters (e.g., oxygen, C/N, and TN) were not apparently extreme compared to other sites. However, the porosity values were the lowest at the P03 site. The effect of porosity and pore size distribution can properly account for shifts in microbiota composition [61,90]. Therefore, porosity (probably influenced by the mixture of sandy and muddy sediments) may explain in part the dramatic difference in composition for site P03. However, the uniqueness of this site remains unexplained.

This study marked proximity to the PRE as the main factor controlling the composition of microbiota in the sediment not only inside the PRE but also at marine sites in the region. Furthermore, we found that the effect is similar in size for bacteria, archaea, and Eukaryota, indicating probable dramatic shifts in microbial activity and hence all biogeochemical processes in the sediment. The samples from different sites were characterized by specific marker populations, which were linked to variations in specific chemical properties of the sediment, particularly C/N ratio and NH_4_^+^ concentration. Nevertheless, microbiota composition at specific sites may be related to factors unique to the site (chronic or transient) that should be further uncovered. In addition, a better understanding may be achieved by considering seasonal variations and the addition of functional information derived from metagenomes. Therefore, monitoring the microbiota composition of PRE may contribute to ongoing efforts for regional control of the marine ecosystem.

## Data Availability

The data presented in this study have been deposited in the NCBI repository, accession number: PRJNA1031164, with the following URL: http://www.ncbi.nlm.nih.gov/bioproject/PRJNA1031164, accessed on 20 June 2024.

## Supplementary Materials

The following supporting information can be downloaded at: https://www.mdpi.com/article/10.3390/microorganisms12071273/s1, Figure S1: Venn diagrams indicating the distribution of unique and shared microbial ASVs between the sites. ASVs with prevalence >10% of samples were included. Only values above 1% are shown. Figure S2: Boxplot presenting the distribution of Shannon H’ index of diversity within each site at each kingdom. Table S1: Coordinates of sampled sites. Table S2: Metadata of physicochemical parameters (based on Zhou Lei Thesis, 2022). Table S3: Multiple regression calculation of an environmental variable NMDS ordination axes (the significant values are highlighted). Table S4: Effect of the site and core depth on sediment microbiota. PREMANOVA factorial test results. Table S5: Effect of the site and core depth on sediment microbiota. The factors were examined by a pairwise multilevel comparison using the Adonis test. Table S6: a. Taxonomy and raw read counts for archaea samples. b. Taxonomy and raw read counts for bacteria samples. c. Taxonomy and raw read counts for Eukaryota samples. Table S7: a. Archaeal markers determined by linear discriminant analysis (LDA) effect size (LefSe) for site and core depth. b. Bacterial markers determined by linear discriminant analysis (LDA) effect size (LefSe) for site and core depth. c. Eukaryote markers determined by linear discriminant analysis (LDA) effect size (LefSe) for site and core depth. Table S8: Correlation between site (a) or core depth (b) and chemical parameters of sediments. Significant ASVs for both site/core depth (based on Table S7) and at least one physiochemical parameter are shown. Significant effects of sediment chemical properties were examined by linear decomposition model (LDM) analysis.
