# Supplementary material for: Exploring the Microbial Mosaic: Insights into Composition, Diversity, and Environmental Drivers in the Pearl River Estuary Sediments
Source: Microorganisms. 2024 Jun 23;12(7):1273. doi: 10.3390/microorganisms12071273 (PMC11279356; doi:10.3390/microorganisms12071273)
Supplement: Supplementary file 1 [file microorganisms-12-01273-s001.zip › Supplementary.pdf]

Supplementary figures and tables.

Table S1: Coordinates of sampled sites

Site name	Bottom depth (m)	Longitude (N)	Latitude (E)
A02	62	21.501	114.252
A01b	41	21.751	114.123
A01	33	22.001	113.998
P03	24	21.997	113.851
P02	16	21.994	113.698

Table S4: Effect of the site and core depth and on sediment microbiota. PREMANOVA factorial test results.

	Factor	F	R^2	P
Archaea	**Site**	5.5577	0.29761	< 0.001
	**Core_Depth**	3.5172	0.09417	< 0.001
	**Site X Core_Depth**	1.929	0.2066	< 0.001
Bacteria	**Site**	8.8792	0.40901	< 0.001
	**Core_Depth**	4.0425	0.09311	< 0.001
	**Site X Core_Depth**	1.6541	0.15239	< 0.005
Eukaryota	**Site**	4.1156	0.2381	< 0.001
	**Core_Depth**	3.684	0.10657	< 0.001
	**Site X Core_Depth**	1.9136	0.22142	< 0.001

Table S5: Effect of the site and core depth and on sediment microbiota. The factors were examined by a pairwise multilevel comparison using Adonis test.

	Site X Site		R^2	F	P	Bonferroni
Archaea	A01b	A01	0.18527	3.6385	0.001	0.01
	A01b	P03	0.16709	3.2098	0.001	0.01
	A01b	P02	0.19382	3.8467	0.001	0.01
	A01b	A02	0.13232	2.44	0.014	0.14
	A01	P03	0.24913	5.3085	0.001	0.01
	A01	P02	0.22696	4.6975	0.001	0.01
	A01	A02	0.34039	8.2568	0.001	0.01
	P03	P02	0.16877	3.2487	0.001	0.01
	P03	A02	0.17867	3.4805	0.001	0.01
	P02	A02	0.28371	6.3373	0.001	0.01
Bacteria	A01b	A01	0.31442	7.338	0.001	0.01
	A01b	P03	0.22471	4.6376	0.001	0.01
	A01b	P02	0.32478	7.696	0.001	0.01
	A01b	A02	0.25382	5.4424	0.001	0.01
	A01	P03	0.24379	5.1581	0.001	0.01
	A01	P02	0.236	4.9424	0.001	0.01
	A01	A02	0.45977	13.617	0.001	0.01
	P03	P02	0.18476	3.626	0.001	0.01
	P03	A02	0.29622	6.7344	0.001	0.01
	P02	A02	0.45397	13.302	0.001	0.01
Eukaryota	A01b	A01	0.18392	3.6059	0.001	0.01
	A01b	P03	0.15063	2.8374	0.001	0.01
	A01b	P02	0.20342	4.0859	0.001	0.01
	A01b	A02	0.12983	2.3872	0.003	0.03
	A01	P03	0.14131	2.633	0.002	0.02
	A01	P02	0.14781	2.7752	0.004	0.04
	A01	A02	0.22092	4.5369	0.001	0.01
	P03	P02	0.1306	2.4035	0.001	0.01
	P03	A02	0.13288	2.4519	0.001	0.01
	P02	A02	0.2087	4.2198	0.001	0.01

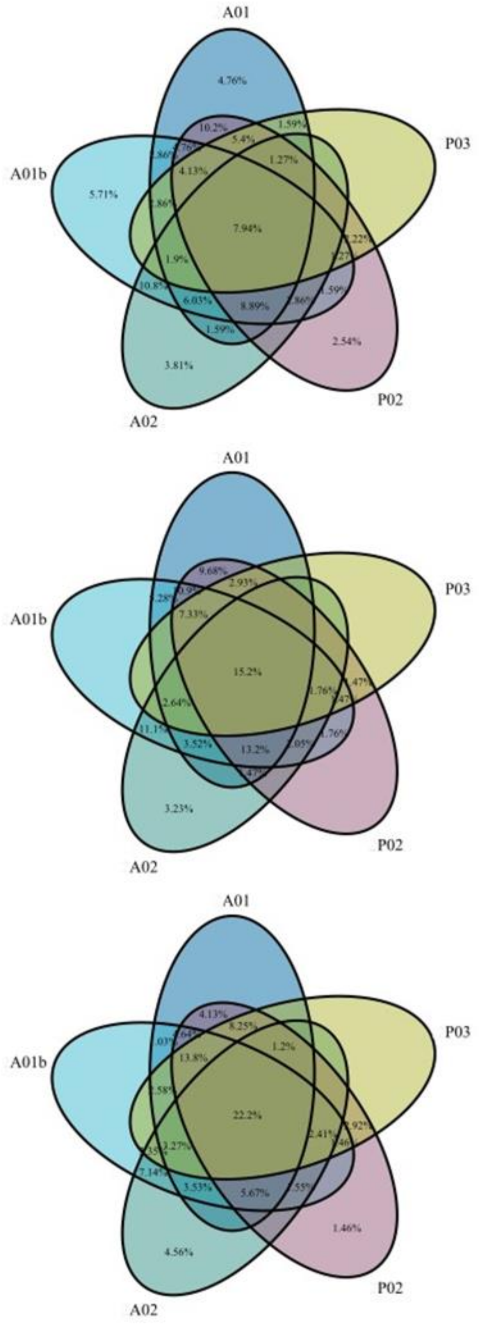

Figure S1: Venn diagrams indicating the distribution of unique and shared microbial ASVs between the sites. ASVs with prevalence >10% of samples were included. Only values above 1% are shown.

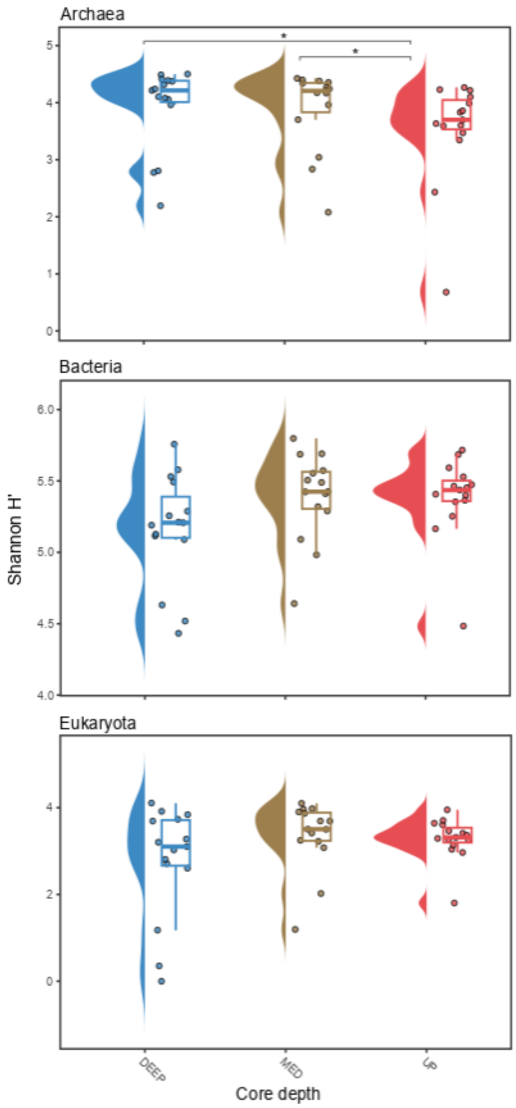

Figure S2: Boxplot presenting the distribution of Shannon H' index of diversity within each site at each kingdom.
